# Information Needs for Opioid Use Disorder Treatment Using Buprenorphine Product: Qualitative Analysis of Suboxone-Focused Reddit Data

**DOI:** 10.2196/68886

**Published:** 2025-06-09

**Authors:** Madhusudan Basak, Omar Sharif, Sarah E Lord, Jacob T Borodovsky, Lisa A Marsch, Sandra A Springer, Edward V Nunes, Charles D Brackett, Luke J Archibald, Sarah M Preum

**Affiliations:** 1 Department of Computer Science Dartmouth College Hanover, NH United States; 2 Department of Computer Science and Engineering Bangladesh University of Engineering and Technology Dhaka Bangladesh; 3 Center for Technology and Behavioral Health Geisel School of Medicine Dartmouth College Lebanon, NH United States; 4 Department of Biomedical Data Science Geisel School of Medicine Dartmouth College Lebanon, NH United States; 5 Department of Psychiatry Dartmouth Health Lebanon, NH United States; 6 Department of Psychiatry Geisel School of Medicine Dartmouth College Lebanon, NH United States; 7 Section of Infectious Disease Department of Internal Medicine Yale School of Medicine New Haven, CT United States; 8 Center for Interdisciplinary Research on AIDS Yale University School of Public Health New Haven, CT United States; 9 Section of Infectious Disease Department of Internal Medicine Veterans Administration Connecticut Healthcare System West Haven, CT United States; 10 Department of Psychiatry Division on Substance Use Columbia University Irving Medical Center and New York State Psychiatric Institute New York, NH United States; 11 Department of Medicine Geisel School of Medicine Dartmouth College Hanover, NH United States; 12 Department of Internal Medicine Dartmouth Hitchcock Medical Center Lebanon, NH United States

**Keywords:** opioid use disorder, medication for opioid use disorder, MOUD, buprenorphine, Reddit, social media, treatment information needs, access, substance use, tapering, patient experience

## Abstract

**Background:**

Buprenorphine is a Food and Drug Administration–approved medication for opioid use disorder. However, individuals with opioid use disorder often report information needs regarding buprenorphine treatment on social media platforms such as Reddit. The field lacks a systematic approach to organizing these data and characterizing treatment information needs that may be unique and unavailable elsewhere.

**Objective:**

In this study, we curated and analyzed large-scale data from social media to characterize self-reported buprenorphine treatment information needs using a qualitative analysis.

**Methods:**

We collected 15,253 Reddit posts from the subreddit r/Suboxone. Following a standard protocol and guidance from clinical experts, we first identified 5 main themes from the data and then manually coded 6000 posts based on these themes. Finally, we determined the most frequently appearing topics within each theme by analyzing samples from each group.

**Results:**

Among the 6000 posts, 2417 (40.3%) contained a single theme, 2160 (36%) contained two themes, and 834 (13.9%) contained three themes. The most frequent topics found across these themes included reports of psychological and physical effects during recovery, complexities in accessing buprenorphine, and significant information gaps regarding medication administration, tapering, and substance use at different stages of recovery. Moreover, self-treatment strategies and peer-driven advice revealed potential rumors and misinformation.

**Conclusions:**

Our findings can guide the design of interventions to improve patient education and communication. They also help address knowledge gaps and misinformation related to treatment. Additionally, the results can support hypothesis generation for future clinical research on medication for opioid use disorder treatment.

## Introduction

Opioid use disorder (OUD) remains a significant public health concern in the United States. More than 81,000 deaths from opioid overdoses were reported in 2022, contributing to more than 700,000 deaths from 1999 to 2022 [1]. Buprenorphine, methadone, and naltrexone are Food and Drug Administration–approved medications for opioid use disorder (MOUDs) and gold-standard treatments for OUD [2-4]. Individuals considering or receiving MOUD treatment often report a range of information needs related to different aspects of treatment [5], including accessing MOUD, medication schedule (timing or dosage), concurrent substance use, unexpected symptoms and side effects, and tapering off MOUD. When unaddressed, these issues can result in noncompliance with treatment, causing delays, discontinuation, use of unverified treatments, and deaths [4,6-8].

Identifying and characterizing the treatment information needs (TINs) of individuals with OUD is a critical first step to designing effective interventions for MOUD treatment induction, adherence, and retention. Traditional approaches to identifying TINs have included surveys, structured interviews, and focus groups that engage directly with patients and providers. Our approach complements these methods by leveraging naturally occurring, self-reported discourse in web-based communities. In this study, we curated a large dataset of Reddit posts, classified the data into granular categories (herein referred to as themes), and defined theme-based characteristics such as frequency, co-occurrence, and patterns.

Social media can help us capture the diversity of TINs of thousands of individuals with lived experiences and the real-world complexity of recovery [9-15]. In the United States, approximately 70% of the population uses social media [16]. What sets social media apart from traditional data sources is the spontaneous, self-reported lived experiences shared by individuals with OUD, a type of data that is not easily obtainable through other data sources such as electronic health records or surveys. This aspect is particularly vital in the context of OUD, a highly stigmatized topic [17-19] where persons with OUD often hesitate to reach out to traditional health care providers to address TINs due to a lack of access, trust, or health equity [20-23].

The current research on identifying MOUD-related TINs on social media has 2 major limitations. First, there is no large, publicly available dataset curated by knowledgeable coders and verified by experts that captures self-narrated discourse on MOUD TINs. Most existing datasets are small, which limits the generalizability of the findings [17,24]. Second, while some studies have explored a set of specific themes, such as seeking advice on physical or psychological effects while tapering or quitting buprenorphine products [25], most existing work focuses on 1 type of MOUD TIN at a time [26-29]. This narrow focus risks overlooking meaningful interconnections among co-occurring concerns. For example, individuals often simultaneously discuss tapering strategies, the timing of transitioning from substances such as fentanyl or heroin to buprenorphine, and how to manage side effects. To develop a more comprehensive understanding of these challenges, it is essential to analyze posts that involve multiple, overlapping themes and reflect the complexity of real-world experiences.

We address this research gap by proposing a theme-driven framework that provides a labeled dataset. A comprehensive analysis is performed on this dataset, covering multiple themes of TINs and capturing Reddit post data from thousands of affected individuals. As a use case of MOUD, we considered buprenorphine products, as they are one of the most widely used and available MOUD treatment options [28].

## Methods

### Overview

**Figure 1:**
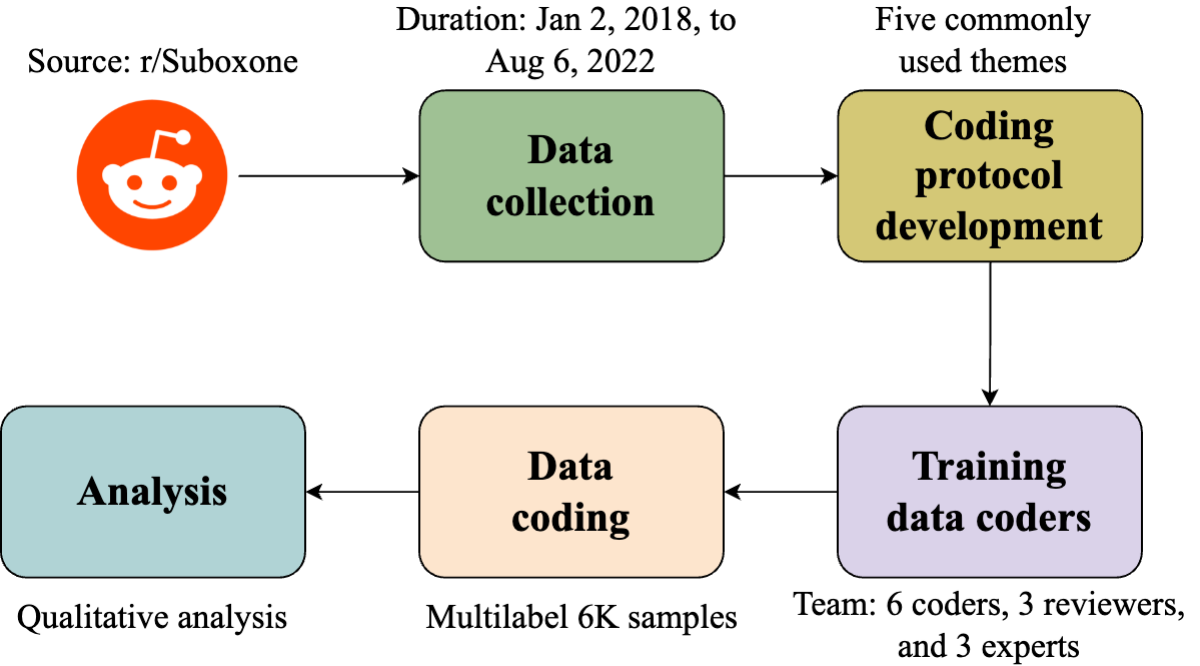
Methodology flow diagram of our framework.

We used a comprehensive methodological framework, encompassing the entire process from selecting data sources to conducting experimental analyses. This framework is built upon existing research and can complement and augment other qualitative, content analysis, and mixed methods research [20,29-32]. After collecting social media posts from the r/Suboxone Reddit community, we randomly selected 6000 posts for further analysis. We identified 5 key themes from a subset of the data and then manually categorized the full data based on these themes, with each post potentially assigned 1 to 3 relevant themes. Finally, we analyzed a subset of posts from each category to identify the most common subthemes (herein referred to as topics) within each main theme. Figure 1 highlights the key steps of our framework.

### Data Collection

Using PRAW and PushShift application programming interfaces [33,34], tools that help researchers access public Reddit data, we collected posts, comments, likes, upvotes, and unique post identifiers from r/Suboxone (41K members). There are other subreddits specific to a MOUD option, for example, r/Sublocade, r/Subutex, and r/Methadone. However, we selected r/Suboxone because (1) Suboxone is one of the most frequently prescribed and available buprenorphine products, (2) r/Suboxone is the largest community among the communities of buprenorphine products, with over 41K members, and (3) this community welcomes other opioid-related discussions too. We followed Reddit policy and the Institutional Review Board protocol of Dartmouth College to collect and postprocess data. Subsequently, we had 15,253 posts available for our study. Due to resource constraints, we randomly chose 6000 posts for manual coding, where each post contained less than 300 words. The length restriction was implemented to ensure a more focused and precise assessment by the coders.

### Develop Data Coding Protocol

After collecting the data, we developed a data coding protocol for identifying themes in TINs, which followed the following steps.

- Step 1: we developed the initial coding protocol. Following a thematic content analysis method, we used an iterative coding process to identify and delineate the themes that emerged from the data. First, authors SMP, MB, and OS used an inductive approach on a subset of 250 sample posts and identified the recurring themes from the data. They also documented the initial definition of these themes. Then, they shared the theme definitions with a relevant subset of examples with experts SEL and JTB. The experts (SEL and JTB) complemented the findings from the inductive analysis with deductive analysis using existing theories or hypotheses related to information-seeking behavior and information needs of individuals considering or undergoing MOUD treatment. Based on the expert feedback, the initial themes were consolidated into 5 main themes. We followed the thematic saturation method to build consensus around the primary themes. Then, these 5 authors (MB, OS, SEL, JTB, and SMP) developed a systematic coding protocol to define and delineate the 5 main themes of TINs.
- Step 2: we revised the coding protocol through iterative data coding. Then, the 5 authors iteratively coded an additional 1250 posts to define the scope and boundaries of each theme and revised the coding protocol to delineate each theme concretely. In this phase, the authors found some samples that neither fall under the 5 primary themes nor show a pattern to call for a new theme. Hence, these outliers were categorized as having a generic “Others” theme. Additionally, the whole process also generated supplementary information for coding (eg, a dictionary of different brand names for buprenorphine products, street names, and variations of different substances, as they are often mentioned in Reddit posts).
- Step 3: triangulation. To ensure the consistency and reliability of the coding protocol, we applied researcher triangulation. Specifically, 3 additional subject matter experts (LAM, SAS, and EVN) conducted an unbiased review of the coding protocol. These experts are addiction researchers, and 2 also practice addiction psychiatry. Specifically, they were asked to review the correctness of the theme definition and labeling, any missing critical themes, and any issues that need clarification. The coding protocol was updated based on their feedback and suggestions.

The resulting themes related to buprenorphine product-specific TINs are accessing buprenorphine (AccBup), taking buprenorphine (TakeBup), experiencing psychophysical effects (Psyphy), co‑occurring substance use (CoSU), and tapering buprenorphine (TapeBup). Some of these themes reflect the early stage of recovery (eg, AccBup and TakeBup), while others reflect the recovery continuum (eg, CoSU and Psyphy). The definitions of these themes are presented in the Results section.

### Large-Scale Data Coding of Posts

We performed a 3-month-long large-scale coding of an additional 4500 samples by a team of 11 members. Three authors (MB, OS, and SMP) formed the reviewer team, the coding team comprised 3 graduate students and 3 undergraduates recruited at the authors’ institution. The coding team underwent rigorous training sessions led by the reviewer team to gain subject matter knowledge. The coders performed 4 rounds of coding spanning over 6 weeks. To maintain the quality of the coding, each sample was independently assigned to 2 different coders. The reviewer team resolved the disagreements and finalized the coding. Authors SEL and JTB independently reviewed 100 samples from the dataset to ensure the quality and consistency of the final dataset.

### Determining Topics Within Themes

To determine the most frequent topics for each theme, we randomly selected at most 50 posts from each theme category. This choice of 50 posts was made as it represents roughly one-fifth of the average number of posts (240 posts) within each theme. For each theme, 2 coders (senior PhD students in computational health who are trained on the subject matter) assigned topics to each selected post independently. After completing their individual coding, the coders met to compare and discuss their topic assignments, collaboratively developed a unified list of topics for each theme, and revised their initial labels accordingly. Finally, they identified and reported the most frequent topics for each theme.

### Analyzing Peer Comments From “TapeBup” and “CoSU-TakeBup” Themed Posts

There are very limited clinical guidelines for patients on TapeBup and CoSU while undergoing MOUD treatment. However, our coded dataset indicates significant interest in these topics within the r/Suboxone community. Therefore, we analyzed a subset of comments from posts that we had coded as “TapeBup” and “CoSU-TakeBup” to surface peer suggestions addressing the TINs related to these themes. For each theme, we randomly selected 10 posts and had 2 coders review the associated comments. They flagged any comments that contained unverified information provided by peers. Finally, the team, in collaboration with experts, discussed these comments to assess the presence of potential rumors and misinformation.

### Ethical Considerations

The research was approved by the Institutional Review Board at the authors’ institution, Dartmouth College (# STUDY00032630). To further ensure privacy, we paraphrased the posts and comments presented in this study to prevent the direct identification of the Reddit users who made the post or the comment, following the standard procedure of researching Reddit data [20,29,31,35].

## Results

### Main Themes of TINs

From our analysis, we identified 5 principal themes that capture distinct aspects of buprenorphine product-specific TINs. Table 1 presents each theme’s name, abbreviation, and a concise description to guide subsequent analyses.

**Table 1:** The main themes are defined here, along with their corresponding abbreviations. These abbreviations have been used throughout the rest of the paper.

Theme name	Description
Accessing buprenorphine (AccBup)	This theme addresses concerns about accessing buprenorphine, for example, challenges with insurance, pharmacies, and health care providers. Identifying these barriers can help understand factors that affect treatment initiation, adherence, and retention.
Taking buprenorphine (TakeBup)	This theme highlights concerns about the treatment regimen for buprenorphine, for example, questions on dosage, timing, and frequency. It emphasizes the potential for misconceptions that may hinder treatment adherence.
Experiencing psychophysical effects (Psyphy)	This theme includes concerns about the physical and psychological effects experienced or anticipated during recovery. It covers both rare and common effects of buprenorphine, examining how these may influence treatment adherence.
Co-occurring substance use (CoSU)	This theme explores concerns related to using other substances during recovery, whether for recreational use or self-medication. It offers insights into individuals’ experiences with substance use alongside buprenorphine treatment.
Tapering buprenorphine (TapeBup)	This theme focuses on concerns about reducing or discontinuing buprenorphine use. It provides insights into self-tapering practices, including reasons, timing, and the effectiveness of tapering strategies.

### Distribution of Themes

In our dataset, we labeled each post with themes evoked from the data. Each theme either appeared as a stand-alone theme or co-occurred with other themes in a post. Some posts contained up to 3 different themes. So, we break down the frequency distribution into 3 groups: posts with 1 theme, posts with 2 themes, and posts with 3 themes. Table 2 presents the distribution of posts among different theme groups. Additionally, 589 of 6000 (9.8%) posts were categorized as “Others,” indicating that they did not have any main theme labels.

**Table 2:** Frequency of the theme combinations. We used abbreviations for each theme, such as AccBup^a^, CoSU^b^, TakeBup^c^, Psyphy^d^, and TapeBup^e^. The ordering of themes inside the theme combinations is chronological and does not carry any positional significance.

Row number		Themes	Frequency, n	Percentage within the theme category, %	Percentage within the whole dataset (N=6000), %
**Posts with a single theme (total=2417, 40.3%)**					
	1	Psyphy	700	29	11.7
	2	AccBup	672	27.8	11.2
	3	TakeBup	527	21.8	8.8
	4	CoSU	281	11.6	4.7
	5	TapeBup	237	9.8	4
**Posts with 2 themes (total=2160, 36%)**					
	6	Psyphy-TapeBup	738	34.2	12.3
	7	TakeBup-Psyphy	391	18.1	6.5
	8	CoSU-TakeBup	335	15.5	5.6
	9	CoSU-Psyphy	274	12.7	4.6
	10	CoSU-TapeBup	105	4.9	1.8
	11	TakeBup-TapeBup	105	4.9	1.8
	12	AccBup-Psyphy	68	3.1	1.1
	13	AccBup-CoSU	64	3	1.1
	14	AccBup-TakeBup	57	2.6	1
	15	AccBup-TapeBup	23	1.1	0.4
**Posts with 3 themes (total=834, 13.9%)**					
	16	CoSU-TakeBup-Psyphy	312	37.4	5.2
	17	CoSU-Psyphy-TapeBup	233	27.9	3.9
	18	TakeBup-Psyphy-TapeBup	142	17	2.4
	19	AccBup-TakeBup-Psyphy	34	4.1	0.6
	20	CoSU-TakeBup-TapeBup	33	4	0.6
	21	AccBup-CoSU-Psyphy	31	3.7	0.5
	22	AccBup-Psyphy-TapeBup	27	3.2	0.5
	23	AccBup-TakeBup-TapeBup	8	1	0.1
	24	AccBup-CoSU-Psyphy	7	1	0.1
	25	AccBup-CoSU-TakeBup	7	0.8	0.1

^a^AccBup: accessing buprenorphine.

^b^CoSU: co-occurring substance use.

^c^TakeBup: taking buprenorphine.

^d^Psyphy: experiencing psychophysical effects.

^e^TapeBup: tapering buprenorphine.

Among the total 6000 posts, 2417 (40.3%) were labeled with only 1 theme (rows 1-5). The frequency distribution of individually labeled themes was uneven, with Psyphy being the most common (700/6000, 11.7%), followed by AccBup (672/6000, 11.2%). TapeBup had the least frequent occurrence, observed in 4% (237/6000) of posts.

Among the total 6000 posts, 2160 (36%) were labeled with 2 themes (rows 6-15). Psyphy-TapeBup (posts labeled with both Psyphy and TapeBup) was the most common, appearing in 12.3% (738/6000) of posts. In contrast, only 0.4% (23/6000) of posts were labeled with AccBup-TapeBup. Conversely, 834 (13.9%) posts were tagged with 3 themes (rows 16-25). Three combinations dominated: (1) CoSU-TakeBup-Psyphy (312/6000, 5.2%), (2) CoSU-Psyphy-TapeBup (233/6000, 3.9%), and (3) TakeBup-Psyphy-TapeBup (142/6000, 2.4%). The remaining 7 combinations contributed to a total of 2.5% (147/6000).

### Findings Surfaced From Thematic Analysis

#### Prevalence of Reporting Psychophysical Effects During Recovery

We observed that Psyphy was a highly prevalent theme (as shown in Table 2). As Psyphy broadly covers both physical and psychological effects, we analyzed the data (1) to separately identify the common physical and psychological effects within self-reported contents of the posts and (2) to determine whether each group of effects has the potential correlation with other themes. For this purpose, we randomly sampled 100 posts from the pool of posts tagged with Psyphy. We found that the psychological effects (eg, anxiety, suicidal thoughts, or anger) are found mostly when the posts also discuss CoSU and TakeBup. Meanwhile, some physical effects, such as puking, sweating, and restless legs, are common when the theme Psyphy co-occurs with the theme TakeBup in a post, whereas other physical effects, such as insomnia, low energy, sneezing, etc, are frequent when Psyphy co-occurs with the theme TapeBup. Meanwhile, withdrawal commonly appears in almost every combination, that is, whether theme Psyphy occurs as a stand-alone theme or co-occurs with any other theme. These findings have implications for clinical and public health research as well as targeted interventions for patient communication and education.

#### Complexities Stemming From Barriers to AccBup

Our thematic analysis reveals different contexts of access barriers to buprenorphine as well as a wide range of complexities stemming from lack of access to buprenorphine (AccBup). For example, row 2 of Table 3 shows examples of individuals seeking treatment asking questions to resolve different access barriers, including accessing telehealth options for treatment, issues with prescription refills at the pharmacy, and insurance coverage. We observed several additional complexities stemming from the lack of access to treatment. Such as considering other buprenorphine formulations (eg, Suboxone to Zubsolv in row 9 in Table S1 in Multimedia Appendix 1), tapering and self-dosing buprenorphine (row 10 of Table S1in Multimedia Appendix 1), self-treatment strategies to manage the associated psychophysical effects (rows 14, 17, and 18 in Table S1 in Multimedia Appendix 1), and considering other substances (eg, oxycodone, kratom, or hydrocodone) to reduce the side effects caused by stopping or lowering the dose of buprenorphine product (rows 16, 19, and 20 in Table S1 in Multimedia Appendix 1).

**Table 3:** Table containing the frequently discussed topics for posts with 1 theme (extended table for posts with multiple themes is shown in Table S1 in Multimedia Appendix 1).

Theme	Commonly discussed topics with the theme	Examples (paraphrased and redacted samples)
**1. Psyphy^a^**		
	Seeking peer’s experiences and advice on psychological or physical effects (eg, tooth problem or shy bladder) experienced while undergoing treatment with buprenorphine products for OUD^b^ treatment.	“After 14 months on 6mg of subs, my teeth are constantly aching. Does anyone else experience this side effect?”
	Seeking information on the reason (eg, why the problem happens only in the morning, why the problem has arisen now, albeit the same usage as before) of a particular side effect (eg, morning sickness, headaches, or agitated feeling) that emerged from the use of buprenorphine products.	“My significant other and I transitioned from fentanyl to Suboxone and today marks our 30th day on it. We've noticed that while we feel fine during the day, both of us experience sneezing, belly cramps, and an unpleasant beginning-of-sickness feeling after a night's sleep. We haven't missed any doses. What could be causing this?”
**2. AccBup^c^**		
	Seeking information on different aspects (eg, how to enroll in the telemedicine service or how much time to wait to obtain the refill) of web-based medication providers (eg, Quick.md, Bicycle Health, or Bupe.me).	“I'm with Bicycle Health now, but I might get dismissed since I can't finish due to home tests. Can I still join Quick.md?”
	Seeking information on pharmacy refill issues (eg, pharmacy discontinued the previous brand, delay in refill by the pharmacy).	“Could someone please inform me about big chain pharmacies that stock the Sandoz brand? The pharmacy I typically visit has recently switched to Alvogen.”
	Seeking information on insurance problems (eg, sudden loss of insurance, whether a particular insurance covers a brand, or a pharmacy not accepting a particular insurance).	“I'm wondering if anyone has information on whether Independent Health provides coverage for generic Suboxone brands?”
**3. TakeBup^d^**		
	Seeking information on the technique to administer (eg, dissolving under the tongue, spitting, or swallowing) the buprenorphine products.	“Where exactly should I place the sub for the gum and cheek method? I'm tired of putting it under my tongue; it feels like half of it goes to waste.”
	Seeking information on the comparative discussion (eg, which one absorbed better or which one is easy to dose) between 2 forms (eg, films vs tablets), brands (eg, name brand vs generic brand), or types (eg, Suboxone vs Sublocade) of buprenorphine products.	“Do the newer Suboxone pills work the same way as the strips, or should they be taken like regular pills? I'm considering switching from strips to pills and wanted to clarify.”
**4. CoSU^e^**		
	Seeking information on the concurrent use of buprenorphine products and controlled substances (eg, kratom, shrooms, or alcohol).	“Any insights on using 'shrooms while in recovery on Suboxone? It's often discussed as potentially beneficial for addicts.”
	Seeking information on starting buprenorphine products to recover from current substance use (eg, fentanyl or morphine).	“I'm dealing with a significant fentanyl dependency. Has anyone here successfully used Suboxone to manage fentanyl addiction? If so, how did you go about it?”
**5. TapeBup^f^**		
	Seeking information on the appropriate technique (eg, the most suitable lower dose to jump, the proper duration to stay on the lower dose before quitting, or the best plan to taper given 50 tablets in hand) to taper buprenorphine products.	“Could someone suggest a tapering plan for quitting 4mg? I've been on subs for 4-5 years.”
	Seeking peer’s suggestions on a particular step or condition (eg, the feasibility of jump offing at 0.4 mg or tapering experience during pregnancy) of a specific tapering strategy (eg, slow taper or fast taper).	“I've learned I'm pregnant and plan to taper my dose, but I'm worried about NAS or losing custody if I don't quit entirely. Has anyone given birth on Suboxone? What was your experience?”

^a^Psyphy: experiencing psychophysical effects.

^b^OUD: opioid use disorder.

^c^AccBup: accessing buprenorphine.

^d^TakeBup: taking buprenorphine.

^e^CoSU: co-occurring substance use.

^f^TapeBup: tapering buprenorphine.

#### Information Gaps Regarding Taking of Buprenorphine Products (TakeBup)

We identified several common types of cases of information gaps related to TakeBup, including techniques to administer the buprenorphine products (eg, dissolving under the tongue, spitting, or swallowing), absorption rate, ease of use, dosing, brand comparison (row 3 in Table 3), how to dose during tapering (row 6 in Table S1 in Multimedia Appendix 1), or after a relapse (row 11 in Table S1 in Multimedia Appendix 1).

#### Aspects of Tapering Buprenorphine Products (TapeBup)

While analyzing single-theme posts that are only labeled with the theme TapeBup, we identified different aspects of tapering as well as different methods of TapeBup products (row 5 in Table 3). While analyzing multithemed posts that contained the TapeBup theme, we identified several aspects of tapering-related information needs. For instance, self-dosing and changing administration methods for tapering (row 6 in Table S1 in Multimedia Appendix 1), seeking strategies to cope with psychophysical effects stemming from tapering, including using alternative treatments and controlled substances (rows 1, 5, 12, 13, and 15 in Table S1 in Multimedia Appendix 1).

#### CoSU While in Recovery

Thematic analysis of posts labeled only with CoSU (row 4 in Table 3) reveals many affected individuals seek information about concurrent use of buprenorphine products and controlled substances (eg, kratom, shrooms, or alcohol) and seek treatment options tailored to their substance dependence history (eg, dependent on fentanyl patch vs heroin). While analyzing posts labeled with other themes in addition to CoSU, we identified several information needs that can impact treatment induction and retention. These include how long to wait to start a buprenorphine product after substance use (row 3 in Table S1 in Multimedia Appendix 1) and use of controlled substances to cope with the psychophysical effects stemming from recovery treatment (row 4, 11, 14, 16, and 19 in Table S1 in Multimedia Appendix 1) or from tapering (rows 5, 12, 15, 17, and 20 in Table S1 in Multimedia Appendix 1).

### Discovering Self-Treatment Strategies

Our analysis also illuminates several self-treatment strategies for which individuals seek information from peers. These include asking questions about self-tapering buprenorphine products and self-dosing different medications to cope with the psychophysical effects of OUD treatment. Table 4 represents some example excerpts of seeking self-treatment strategies. The comments in these posts reveal peer-suggested self-treatment strategies for different themes.

**Table 4:** Examples of self-treatment strategies.

Self-treatment strategy	Examples (paraphrased and redacted samples)
Self-tapering	“Could someone suggest a tapering plan for quitting 4mg?” “Seeking advice on quitting subs using kratom.”
Self-dosing	“Is it okay to take 2mg of subs now?” “I only have a few 5mg hydrocodones. Can taking those help alleviate the current diarrhea and chills I'm experiencing?” “Clonadine can be helpful if you're getting withdrawal symptoms.”

### Analyzing Peer Comments From “TapeBup” and “CoSU-TakeBup” Themed Posts

Our analysis found peers offering advice based on their individual experiences, which can vary greatly from person to person, deviating from official guidelines. For example, even though it is advised not to take buprenorphine immediately after taking opioids (depending on the specific scenario, it is recommended to wait from 12 to 36 hours [36]), a peer provided the following (paraphrased) response when asked how long to wait to take Subutex (a buprenorphine product) after using oxycodone (an opioid). Thus, peers can inadvertently provide advice that is contrary to established clinical guidelines.

You don't need to wait. I attempted the same approach a while back, and buprenorphine simply blocks the effects of other opioids.

Peer suggestion

Our analysis also surfaced several rumors [37-40], that is, suggestions that cannot be clinically verified, for example, tapering guidelines and regimen to quickly taper off buprenorphine products. Following is an example paraphrased post.

I need help doing a rapid taper. 5 years ago, I was at 16mg and have tapered to 2mg. Within 5 days ago I am down to 1.5mg. How can I be done with it fast?

Several comments in this post and similar posts contain peer suggestions on different aspects of tapering. Although these suggestions are often provided in good faith, they are challenging to verify clinically and are thus considered rumors. It should be noted that while not all rumors are harmful [41], some may contain potentially harmful information.

As another example, individuals with OUD might opt for alternative and unverified treatments, discontinue prescribed medications, or delay seeking professional help due to treatment misinformation [42]. Following is a paraphrased excerpt (row 10 in Table S1 in Multimedia Appendix 1) where an individual has decided to use kratom to taper down Suboxone by self-decision. However, kratom is not clinically prescribed to be used during tapering the buprenorphine products.

Hello, seeking advice on quitting subs using kratom. Can I transition directly, or should I taper off subs while starting kratom?

In another post (row 4 in Table 3), the individual intended to use ‘shrooms (the slang wording meaning psilocybin mushrooms, a controlled substance [43]) during the recovery as they found peers discussing the positive sides of this hallucinogen, although using such products is not recommended during MOUD treatment.

Any insights on using 'shrooms while in recovery on Suboxone? It's often discussed as potentially beneficial for addicts.

## Discussion

### Principal Findings

#### Overview

In this study, we applied our theme-driven framework to curate a large dataset coded with multiple themes and perform a thorough analysis involving individual and co-occurring themes. To our knowledge, this is the largest dataset on patient-reported TINs for buprenorphine products for OUD treatment. Our analysis surfaces common information needs, knowledge gaps, and misperceptions that adversely impact initiation and adherence to OUD treatment using buprenorphine products. These findings complement traditional methods such as surveys and interviews by revealing critical gaps in OUD treatment and highlighting ways to improve education and communication with patients.

#### Inform Research on Patient Education and Patient-Provider Communication

Our results surface information gaps across different stages of treatment, such as treatment initiation and tapering, as well as different relevant events, for example, CoSU and the emergence of new treatment options. These findings can provide insights into effectively communicating with patients, providers, recovery coaches, and peer support providers about critical information gaps and how to address them. Effective patient education [44-46] and patient-provider communication [47,48] have become essential parts of modern health care research. Importantly, our findings also underscore the need to consider the therapeutic alliance, the collaborative and trusting relationship between patients and treatment providers, as a foundational element in communication and treatment outcomes. The concerns and questions patients share in web-based platforms reflect both unmet information needs and the dynamics of trust, empathy, and shared decision-making that characterize this alliance. By identifying when patients turn to peer communities rather than health care providers, our findings reveal opportunities to strengthen this alliance through timely, empathetic, and proactive communication. Our findings can also support efforts to proactively address concerns that might impact treatment induction, adherence, and retention, for example, addressing concerns about tapering and long-term use of buprenorphine to avoid tapering and self-dosing, addressing information gaps about medication administration to reduce challenges associated with perceived psychophysical effects resulting from buprenorphine products. These insights can help explain when and why people deviate from clinical guidelines, thereby informing the design of tailored informational interventions to reduce the deviation. Understanding and supporting the patient-provider relationship can also shed light on how trust affects these decisions. Further analysis of such discourse can improve the understanding of stigma and knowledge gaps associated with recovery treatment in a community-informed way.

#### Designing Interventions to Address Treatment-Related Rumors and Misinformation

As discussed in the results sections, there are several critical rumors [38,49] and misinformation [19,50-53] related to treatment that can impact health safety and treatment outcomes. These include false beliefs about using alternative treatments, self-dosing with buprenorphine, and concerns about long-term buprenorphine use. The negative impact of misinformation was also mentioned by existing research [19,52,53]. However, further research is needed to identify the prevalence of such misinformation among different patient populations and geographic locations so that interventions can be designed to address those. Another direction can be developing digital informational interventions to mitigate the effect of potentially harmful information, as demonstrated in research on vaccine hesitancy and lack of adherence to clinical guidelines for infectious diseases.

#### Generate Hypotheses for Clinical Research and Public Health Research

Analyzing such a large-scale, patient-generated discourse can also enable clinical researchers to generate hypotheses [54,55]. For example, they can investigate the link between a prevalent self-reported psychophysical effect and the corresponding buprenorphine product, examine clinically supervised strategies to cope with withdrawal and severe psychophysical effects, and evaluate the effectiveness of different tapering approaches based on the patient’s medical and substance use history. Additionally, our data reveal several cases to capture patients’ lived experiences in a community-informed way to increase treatment adherence and retention, for example, addressing concerns about coping with the psychophysical effects of treatment, long-term effects of treatment, and access barriers to treatment. These types of observations can inform the design of tailored patient-centric studies for public health and addiction researchers. Further, linguistic analysis of the peers’ comments in the posts can generate insights to improve patient-provider communication and interaction with peers and recovery coaches, as well as research on addressing stigma.

#### Complementing Existing Sampling-Based Thematic Analyses

Our dataset and analysis are valuable for analyzing different aspects of OUD treatment due to their large sample size. Although platforms such as Reddit offer a valuable source of real-world spontaneous data for OUD [56,57] or substance use disorder research [58], these analyses often use a limited number of samples to identify prevalent topics, which constrains the generalizability of findings and does not leverage the potential of the data. The limited sample size used by these approaches also impedes the application of computational models to gather rich user-generated content from a broad demographic. In contrast, our extensive dataset, comprising 6000 posts contributed by 3372 unique individuals with OUD, offers a robust resource for identifying frequently observed TINs related to OUD treatment using buprenorphine products. It provides a solid foundation for developing new natural language processing models for OUD and substance use disorder research in a community-informed way [59,60]. Furthermore, existing datasets are often not publicly accessible, limiting reproducibility. We plan to release our dataset to promote future research following Reddit’s data-sharing policies.

### Limitations

We focused on Reddit data, which are subject to gender and age biases—predominantly used by individuals aged between 18-49 years [61] and by male individuals [62]. Meanwhile, the list of themes we considered here is deemed important and significant by experts. However, it is not exhaustive, and there may be other noteworthy themes not included. An alternative conceptualization of the themes could also lead to different results. Again, a subreddit focusing on Subutex- or methadone-based OUD treatment might yield different themes.

Moreover, our dataset cannot provide information on aspects that individuals do not self-disclose, such as using nonprescribed buprenorphine products for treatment. Additionally, we lack data on how many individuals are already in treatment or considering starting treatment unless they self-disclose. This reliance on self-reported data presents an additional limitation, as we have no independent means of verifying whether Reddit users meet clinical criteria for OUD, are currently receiving buprenorphine treatment, or are accurately representing their experiences. Without verification or citation from within the subreddit (eg, r/Suboxone) or related forums, the authenticity of such claims remains uncertain.

We also used Reddit posts containing less than 300 words, but future research can use longer texts to obtain more insights. Additionally, our analysis focused primarily on the content of posts rather than the associated comment threads. We selectively analyzed comments only for 2 themes, “TapeBup” and “CoSU-TakeBup,” to illustrate the nature of peer-shared advice and surface potential misinformation. A more comprehensive and systematic analysis of comment threads across all themes was beyond the scope of this study, but represents an important direction for future work to better understand information gaps, peer interpretations, and emergent self-treatment strategies.

### Conclusions

Our primary purpose was to identify the TINs of individuals considering or undergoing OUD treatment using buprenorphine products on Reddit. The thematic analysis is a valuable resource for gaining deeper insights into increasing treatment induction, adherence, and retention while paying attention to patients’ sense of autonomy and concerns about long-term treatment’s safety, effectiveness, and accessibility. Overall, the curated dataset can contribute to examining treatment safety, effectiveness, and accessibility for individuals with OUD. Moving forward, this work can be a basis for several potential future research studies. We can leverage the dataset and framework to gather insights on sociocultural, behavioral, and health-related questions for minority health and health disparities following the National Institute on Minority Health and Health Disparities Research Framework [63]. Although Reddit is an anonymous platform, such information is sometimes self-disclosed by individuals seeking treatment [64,65]. While this paper mostly focuses on analyzing the posts, further analysis of the comments associated with these posts can reveal additional insights. Further, future research can use this framework in other subreddits and online platforms (eg, YouTube or Facebook) to streamline the TIN identification process and potential interventions to address these TINs.

## Appendix

Multimedia Appendix 1 Table containing the frequently discussed topics for posts with 2 and 3 themes.

## Abbreviations

AccBup: accessing buprenorphine
CoSU: co-occurring substance use
MOUD: medication for opioid use disorder
OUD: opioid use disorder
Psyphy: experiencing psychophysical effects
TakeBup: taking buprenorphine
TapeBup: tapering buprenorphine
TIN: treatment information need
