# Supplementary material for: Information Needs for Opioid Use Disorder Treatment Using Buprenorphine Product: Qualitative Analysis of Suboxone-Focused Reddit Data
Source: J Med Internet Res. 2025 Jun 9;27:e68886. doi: 10.2196/68886 (PMC12186000; doi:10.2196/68886)
Supplement: Multimedia Appendix 1 [file jmir_v27i1e68886_app1.docx]

**Table S1.** Table containing the frequently discussed topics for posts with two and three themes. We used abbreviations for each theme, such as *AccBup*: Accessing buprenorphine, *CoSU*: Co-occurring Substance Use, *TakeBup*: Taking buprenorphine, *Psyphy*: Experiencing Psychophysical Effects, *TapeBup*: Tapering buprenorphine, *AccBup-CoSU-TakeBup*: Combination of Accessing buprenorphine, Co-occurring Substance Use, and Taking buprenorphine. The ordering of themes inside a theme combination (e.g., *Psyphy-TapeBup*) is chronological and does not carry any positional significance.

|  | Row No. | Theme | Commonly Discussed topics with the theme | Examples (paraphrased and redacted samples) |
| --- | --- | --- | --- | --- |
|  |  |  |  |  |
| **Posts with two themes** |  |  |  |  |
|  | 1 | Psyphy-TapeBup | Seeking information on physical or psychological effects (e.g., withdrawal, constipation, anxiety) while tapering or quitting buprenorphine products. | I'm on day 9 of Suboxone withdrawal, feeling hazy. How much longer will this last? I quit at 4mg. |
|  | 2 | TakeBup-Psyphy | Seeking information on changing the brand (e.g., name brand to generic brand) or type (e.g., Suboxone to Subutex) of buprenorphine products due to the side effects (e.g., feeling tired, affecting stomach) caused by the current brand or type. | Subs helped me be present for my family, but they come with overwhelming depression, possibly due to naloxone. Does switching to Subutex or Zubsolv, known to be milder, improve this? Any personal experiences? |
|  | 3 | CoSU-TakeBup | Seeking information on the proper time gap to switch from substance (e.g., fentanyl, heroin) use to buprenorphine products. | I relapsed today. Can I take Subutex tomorrow, considering it hasn't fully left my system? |
|  | 4 | CoSU-Psyphy | Seeking information on using substances (e.g., alcohol, oxycodone, Percocet) while on buprenorphine products, and the resultant side effects (e.g., feeling sick, feeling shit, withdrawal). | Taking 2mg subs for 3 years, is it okay to have a glass of wine without getting sick? |
|  | 5 | CoSU-TapeBup | Seeking information on using substances (kratom, Imodium, etc.) during tapering a buprenorphine products. | Hello, seeking advice on quitting subs using kratom. Can I transition directly, or should I taper off subs while starting kratom? |
|  | 6 | TakeBup-TapeBup | Seeking information on the technique to administer buprenorphine products (e.g., efficiency of diluting into the water, way to cut the strips perfectly) during tapering. | A few mentioned volumetric dosing during sub-2mg taper. Can you explain this approach? |
|  | 7 | AccBup-Psyphy | Seeking information on managing physical or psychological effects (e.g., withdrawal, feeling sick) due to running out early on buprenorphine products for different reasons (e.g., taking extra doses, losing some tablets/pills). | A few days ago, I mentioned running out of my 6mg/day Subutex prescription. It's now around day 6, and I feel terrible. Can the ER assist? I ran out much earlier than expected, and I can't refill for a week. |
|  | 8 | AccBup-CoSU | Seeking information on getting a prescription/refill of buprenorphine products after relapse or recreational use of a specific substance (e.g., kratom, oxycodone, benzos). | I'm concerned my doctor might discontinue my Subutex prescription if there are Ritalin and benzos in my system without his prescription. |
|  |  |  | Seeking induction strategies to start OUD treatment using buprenorphine products from specific substance dependency (e.g., fentanyl, oxycodone) | I must quit kratom, I can't continue. Suboxone seems like the solution, and I need it urgently. Quick.md has positive reviews, but does anyone know if they prescribe Suboxone for kratom addiction? |
|  | 9 | AccBup-TakeBup | Seeking information on the individuals’ experience with the changed brand (e.g., name brand to generic brand) or changed medication (e.g., Suboxone to Zubsolv) due to the unavailability of the regularly used brand/medication for different reasons (e.g., pharmacy not storing the current brand, insurance does not cover the current brand/medication, the health provider changes the brand/medication) | Has anyone here experimented with or is presently using the Butrans patch? I'm contemplating using it since my insurance won't cover Belbuca. Does it adhere reliably for the full 7 days? How effective is it for pain relief? Thanks, everyone! |
|  | 10 | AccBup-TapeBup | Seeking information on a suitable tapering plan for a buprenorphine product use as they anticipate the next prescription refill for the buprenorphine product will be unavailable (e.g., due to the pharmacy discontinuing the medication brand, an individual does not have insurance/money to pay for the next refill). | Today, I collected my prescription which costs $300 (approx..). I can't afford this amount. I think it's time to taper. I intend to reduce from 1.5 strips to 1 for 2 weeks, then to half, and so on. Is this tapering pace too rapid? |
| **Posts with three themes** |  |  |  |  |
|  | 11 | CoSU-TakeBup-Psyphy | Seeking information on a particular strategy (e.g., a particular time gap, a particular dosing schedule) to transition (for the first time or after a relapse) from substance use (e.g., fentanyl, heroin) to OUD treatment with buprenorphine products, and the corresponding side effects (e.g., withdrawal, sweating, stomach cramp). | Took 2, 8mg strips daily for 3 days. Drank and used heroin last night. Wondering if I should wait 24+ hours to take Suboxone or if I can take it sooner to avoid withdrawal discomfort. |
|  | 12 | CoSU-Psyphy-TapeBup | Seeking information on the use of substances (e.g., kratom, Adderall, clonazepam) to get rid of the side effects (e.g., withdrawal, body aches) that emerged while tapering or quitting buprenorphine products. | Has anyone used kratom for Suboxone withdrawal? If yes, I'd appreciate hearing your experiences. I've been trying it during my first week off Suboxone, but I'm feeling uncertain about it. |
|  | 13 | TakeBup-Psyphy-TapeBup | Seeking information on tapering or quitting one buprenorphine product due to its side effects (e.g., tiredness, feeling shitty) and switching to another buprenorphine product (e.g., Suboxone to Sublocade, Subutex to Suboxone). | I've been taking 2-8mg of Suboxone since September. It's made me consistently tired and unmotivated. How have others successfully stopped using it? I've discussed Sublocade with my doctor, and it seems like the best choice for me now. |
|  | 14 | AccBup-TakeBup-Psyphy | Seeking information on the side effects (e.g., withdrawal, stomach pain, lethargy) caused by the change of buprenorphine brand (e.g., name brand to generic brand) or form (e.g., tablets to films), or medication type (e.g., Subutext to Suboxone) due to a logistic barrier (e.g., insurance issue, pharmacy supplying a different brand, intentional change by the health provider) | I've been using Aquestive/Indivior 4mg twice daily, but the local pharmacy is shifting to Alvogen's generic strips. I've mostly found negative feedback about Alvogen. My primary worry is potential withdrawal symptoms. Can it cause precipitated withdrawal? |
|  | 15 | CoSU-TakeBup-TapeBup | Seeking information on the proper time gap to start taking buprenorphine products for the first time after the use of substances (e.g., kratom, heroin), with an intent to use buprenorphine product for a short period of time and then taper it rapidly. | It's been around 8 hours since I stopped using kratom. Is it okay to take 2mg of subs now? I'm aiming for a two-week rapid taper to eventually stop everything. |
|  | 16 | AccBup-CoSU-Psyphy | Seeking information on the use of substances (e.g., oxycodone, kratom, hydrocodone) to reduce the side effects caused by the sudden stop of buprenorphine product use due to a logistic barrier (e.g., running out early, insurance problem). | I've been on Suboxone for a very extended period, taking 24mg daily. Now, I'm completely out of it. I only have a few 5mg hydrocodones. Can taking those help alleviate the current diarrhea and chills I'm experiencing? |
|  | 17 | AccBup-Psyphy-TapeBup | Seeking information on the physical or psychological side effects (e.g., withdrawal, anxiety, insomnia) while doing a taper due to a logistical barrier (e.g., running out early, insurance expiration, high medication cost, sudden unavailability of health provider). | My boyfriend used to take 8mg of Suboxone daily for years. When he lost insurance a month ago, he had to quit. His last dose was around 6/20, and now it's 7/11, but he's still experiencing severe withdrawals - insomnia, skin discomfort, vomiting, and weakness. |
|  | 18 | AccBup-TakeBup-TapeBup | Seeking information on the effective way (e.g., cutting medicine, volumetric dosing) to divide a high-strength medication (e.g., 8 mg strips) to a lower dose (e.g., 0.25 mg) while doing a taper due to a logistic barrier (e.g., insurance issue, run out early). | I was given excessive 20mg daily dosage, abruptly stopped in the 5th month due to insurance issues. My plan was to stabilize and then gradually reduce by 0.25mg, with a week between reductions. I've heard that the buprenorphine distribution on the strips isn't uniform. Is volumetric dosing the better option? |
|  | 19 | AccBup-CoSU-TapeBup | Seeking information on the use of substances (e.g., kratom) while doing a taper due to a logistic barrier (e.g., insurance issue, run out early). | My doctor stopped my autopay, leaving me with a high bill I can't afford right now. I'm on day 8 of quitting an 8mg three times a day medication cold turkey, and it's been incredibly challenging. I tried using kratom, but it hasn't provided any relief. Any suggestions? |
|  | 20 | AccBup-CoSU-TakeBup | Seeking information on the proper way (e.g., the proper time gap between the last substance use and the next buprenorphine dosing) to start the buprenorphine product after a forced relapse because of the temporary unavailability of the buprenorphine product due to a logistic barrier (e.g., running out early, insurance problem). | After my final buprenorphine dose on Tuesday, I had to switch to oxycodone due to the pharmacy's stock shortage. How much time must I wait before resuming the subs? |
